# Synergistic Effects of Type D (“Distressed”) Personality Traits on Coronary Microvascular Function in Male Physicians With Occupational Burnout: A Cross‐Sectional Study

**DOI:** 10.1161/JAHA.125.041273

**Published:** 2025-07-29

**Authors:** Roland von Känel, Paul Lodder, Sarah A. Holzgang, Mary Princip, Andreas A. Giannopoulos, Ronny R. Buechel, Sinthujan Sivakumar, Claudia Zuccarella‐Hackl, Aju P. Pazhenkottil

**Affiliations:** ^1^ Department of Consultation‐Liaison Psychiatry and Psychosomatic Medicine University Hospital Zurich, University of Zurich Zurich Switzerland; ^2^ Department of Methodology and Statistics, Department of Medical and Clinical Psychology, Center of Research on Psychological Disorders and Somatic Diseases (CoRPS) Tilburg University Tilburg The Netherlands; ^3^ Cardiac Imaging, Department of Nuclear Medicine University Hospital Zurich, University of Zurich Switzerland

**Keywords:** burnout, cardiovascular disease, coronary flow reserve, psychological stress, sympathetic nervous system, type D personality, Autonomic Nervous System, Mechanisms, Risk Factors

## Abstract

**Background:**

Psychosocial factors like type D personality (TDP) and occupational burnout are associated with increased coronary artery disease risk. This cross‐sectional study investigated a potential mechanism linking TDP, characterized by negative affectivity (NA) and social inhibition (SI), to coronary microvascular function in male physicians and examined burnout's potential moderating role through a balanced, stratified design.

**Methods:**

Sixty male physicians, evenly divided into burnout and nonburnout groups, underwent positron emission tomography to measure endothelium‐dependent coronary flow reserve (CFR) during the cold pressor test and endothelium‐independent CFR during adenosine‐induced hyperemia. TDP effects were primarily analyzed using a dimensional approach based on continuous NA and SI scores and their interaction, adjusting for physical activity and Systematic Coronary Risk Evaluation 2 or age and metabolic syndrome factors.

**Results:**

Fourteen participants (23.3%) exhibited TDP (NA and SI scores ≥10), with 92.9% also experiencing burnout. Moderator analysis was limited by the high co‐occurrence of burnout and TDP. A significant positive NA×SI interaction was associated with increased endothelium‐dependent CFR, independent of burnout, physical activity, and either Systematic Coronary Risk Evaluation 2 (*P*=0.044, partial η^2^=0.074) or age and metabolic syndrome factors (*P*=0.030, partial η^2^=0.087). CFR increased with higher SI scores when NA was high, and with higher NA scores when SI was high. This effect was mediated by peak rate–pressure product, an indicator of sympathetic stimulation.

**Conclusions:**

TDP components may synergistically affect coronary microvascular function via heightened sympathetic activity. Exaggerated CFR during sympathetic stimulation may reflect a compensatory or early‐stage response, potentially indicative of maladaptive hyperemia and future microvascular impairment.

**Nonstandard Abbreviations and Acronyms**
CFR: coronary flow reserve
CPT: cold pressor test
MBF: myocardial blood flow
MetS: metabolic syndrome
NA: negative affectivity
PHQ‐9: Patient Health Questionnaire‐9
RPP: rate–pressure product
SCORE2: Systematic Coronary Risk Evaluation 2
SI: social inhibition
TDP: type D (“distressed”) personality

**Clinical Perspective**

**What Is New?**

- The interaction of negative affectivity and social inhibition, the core traits of type D personality, is associated with increased endothelium‐dependent coronary flow reserve during sympathetic stimulation in male physicians.

**What Are the Clinical Implications?**

- Elevated coronary flow reserve in response to sympathetic activation may reflect an early maladaptive vascular response in individuals with type D personality traits, potentially increasing long‐term cardiovascular risk.
- Screening for type D personality traits and addressing emotional distress and social inhibition may help identify individuals at risk for coronary microvascular dysfunction and inform preventive strategies in clinical practice.

Psychosocial stress is a significant predictor of morbidity and death in coronary artery disease (CAD), comparable with traditional cardiovascular disease (CVD) risk factors.^1^ Interest is growing in the complex relationship between psychological factors, including personality traits, and poor cardiovascular health, as well as the underlying mechanisms.^2^ Type D (distressed) personality (TDP), characterized by negative affectivity (NA) and social inhibition (SI), predisposes individuals to frequent negative emotions and limited self‐expression in social interactions.^3^ A meta‐analysis of prospective studies in patients with CVD linked TDP to an increased risk of major adverse cardiovascular events,^4^ though debates continue over the best measurement approach of TDP effects.^4^, ^5^ Particularly, the continuous method, incorporating NA, SI, and their interaction, may be more reliable than the historical dichotomous approach (high scores in both NA and SI), which often overestimates the risk posed by TDP when only NA or SI is driving the outcome.^4^, ^6^

Behavioral and physiological mechanisms underlie cardiovascular risks in TDP, including low physical activity, metabolic changes, and endothelial dysfunction.^7^, ^8^, ^9^, ^10^, ^11^ With almost 50%, TDP is twice as prevalent in myocardial infarction with nonobstructive CAD as in obstructive CAD.^9^, ^12^ In myocardial infarction with nonobstructive CAD, endothelial dysfunction and coronary microvascular disease often lead to ischemia and necrosis.^13^ Coronary microvascular function is assessed using positron emission tomography–derived coronary flow reserve (CFR), defined as the ratio of myocardial blood flow (MBF) during maximal coronary vasodilation to resting MBF, measured with pharmacological or physiological stimuli.^14^ The cold pressor test (CPT) activates sympathetic pathways and stimulates endothelium‐dependent hyperemic MBF.^15^ Men with TDP were shown to exhibit stronger cardiac sympathetic responses to CPT, evidenced by a shorter preejection period, than women with TDP or individuals without TDP.^16^ While reduced CFR is a marker of CAD severity and major adverse cardiovascular event risk,^17^ exaggerated CFR in TDP with the CPT might reflect sympathetically driven increases in the rate–pressure product (RPP), signaling heightened global MBF.^18^ Such a response might indicate maladaptive hyperemia, where elevated blood flow velocity increases coronary shear stress, potentially leading to vascular remodeling and endothelial dysfunction over time.^19^

Age may additionally modulate TDP's impact, with younger patients (aged <70 years) experiencing higher major adverse cardiovascular event risks.^20^ Occupational stress and burnout, resulting from prolonged job stress,^21^ are common in health care workers,^22^, ^23^, ^24^ particularly physicians,^25^ and may further increase cardiovascular risk. Potential mechanisms linking burnout to coronary microvascular dysfunction include elevated systemic inflammation,^26^ sustained autonomic dysregulation, characterized by increased sympathetic activity and reduced parasympathetic tone,^26^ and adverse health behaviors.^27^ TDP is positively associated with burnout symptoms,^22^, ^23^, ^24^ and burnout has been associated with an increased CAD risk^28^ and all‐cause death in younger individuals.^29^ These patterns may imply that individuals with TDP or burnout experience heightened disease risk earlier in life.^4^, ^29^

This study investigated the relationship between TDP and coronary microvascular function in male physicians exposed to varying levels of professional stress. To our knowledge, the association between TDP and CFR has not been previously examined. This study addresses this gap by exploring a potential link, extending prior research on cardiovascular biomarkers related to TDP. Importantly, we focused on CFR as an organ‐specific and pathophysiologically proximal marker of cardiovascular dysfunction, moving beyond systemic indicators such as inflammatory markers. Additionally, whereas previous studies typically assessed TDP categorically, we primarily adopted a dimensional framework, analyzing NA and SI as continuous traits along with their interaction, an approach increasingly supported by recent evidence.^6^, ^30^ Secondary categorical analyses were performed for comparison with prior studies.

We specifically hypothesized that the interaction between NA and SI would synergistically be associated with increased endothelium‐dependent CFR independent of age, physical activity, and metabolic factors on this relationship. We further hypothesized that this association would be mediated by sympathetic stimulation, as reflected by peak RPP during the CPT. Furthermore, we examined whether burnout potentially moderates the association between TDP and CFR through a balanced stratified design. Figure 1 depicts a directed acyclic graph illustrating the hypothesized relationships among TDP, relevant covariates, burnout, RPP, and CFR.

**Figure 1:**
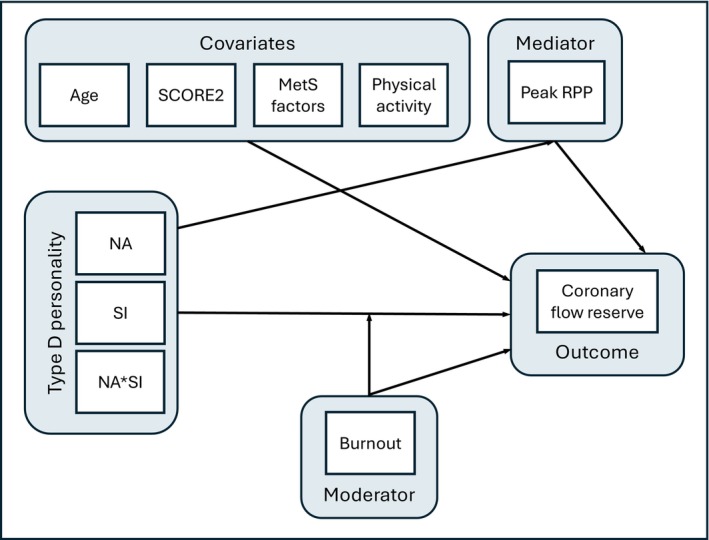
Hypothesized pathways linking TDP to coronary microvascular function. Directed acyclic graph illustrating the hypothesized associations between TDP, its components—NA, SI, and their interaction (NA×SI)—and CFR. In line with the dimensional approach used in this study, TDP is represented as the interaction between NA and SI, rather than NA or SI alone. Peak RPP during the cold pressor test is modeled as a mediator. Age, SCORE2, MetS factors, and physical activity are included as covariates. Burnout (yes/no) is considered a potential moderator of the TDP–CFR association. CFR indicates coronary flow reserve; MetS, metabolic syndrome; NA, negative affectivity; RPP, rate–pressure product; SCORE2, Systematic Coronary Risk Evaluation 2; SI, social inhibition; and TDP, type D personality.

## Methods

### Study Participants and Design

The data that support the findings of this study are available from the corresponding author on reasonable request. The study received approval from the Zurich State Ethics Committee, Switzerland (BASEC‐Nr. 2018‐01974), and all participants provided written informed consent. Between September 2019 and December 2021, this cross‐sectional study recruited 60 male physicians from Switzerland through various channels to examine the impact of burnout on cardiovascular health. Participants were evenly divided into a burnout group (N=30) and a healthy control group (N=30). To ensure balanced group sizes, a quota sampling approach was applied, where recruitment continued until the required number of participants meeting the burnout and control criteria was achieved.

Prior institutional research^31^ implied that 23 participants per group were sufficient to detect a group difference in adenosine‐induced hyperemic MBF with 0.80 power for a Cohen's d of 0.85, typically considered a large effect. For the present analysis of a relation between TDP and CFR, and the moderation of this relationship by burnout status, a power analysis for linear regression indicated that a sample size of N=60 would detect a medium‐to‐large effect (partial η^2^=0.12) with a power of 0.80. Due to the deliberate application of the quota sampling strategy, fewer participants met the categorical TDP criteria than the burnout criteria. Our primary aim was to analyze TDP dimensionally, an approach recommended in the TDP literature.^4^, ^6^ Although we also intended to examine burnout as a potential moderator and sought balanced representation through quota sampling, the high co‐occurrence of burnout and TDP ultimately limited the feasibility of this preplanned analysis.

To reduce measurement bias, validated and standardized instruments were used for psychometric assessments. Eligibility was determined through a telephone screening conducted with group assignment based on the Maslach Burnout Inventory–Human Services Survey^32^ and the Patient Health Questionnaire‐9 (PHQ‐9).^33^ In total, 143 potential subjects were screened, 60 of whom were included in the study. Reasons for exclusion were either that inclusion criteria were not met, exclusion criteria were present, or there was no longer interest in participating in the study. The burnout group required emotional exhaustion scores ≥27 or depersonalization ≥10 (minimum emotional exhaustion ≥20), while the control group required emotional exhaustion <16 and depersonalization <7.^25^ Additional criteria for the burnout group included a PHQ‐9 score ≤14 (moderate depressive symptoms)^33^ and job‐related stress lasting at least 6 months,^34^ while the control group required PHQ‐9 scores ≤10 (mild depressive symptoms).^33^ To minimize selection bias, groups were actively matched by age (within 5 years), body mass index (within 5 kg/m^2^), and family history of early CVD. If an imbalance between the groups arose during recruitment, targeted efforts were made to identify and enroll physicians with the necessary characteristics to maintain comparability. Both groups required participants to be nonsmokers for at least 5 years and aged between 28 and 65 years. This age range was chosen because, in Switzerland, most physicians begin practicing medicine no earlier than around age 28, and 65 marks the official retirement age.

Exclusion criteria for both groups included a history of clinically diagnosed depression, a previous episode of burnout, or clinically diagnosed CVD. Specifically, a history of CAD (eg, myocardial infarction, percutaneous coronary intervention, or bypass surgery) was an exclusion criterion, to avoid confounding by structural coronary obstruction and to focus specifically on microvascular function. A history of prior burnout was an exclusion criterion in the control group to avoid misclassification by excluding individuals with potential residual symptoms or altered stress reactivity despite current remission. In the burnout group, exclusion of individuals with recurrent burnout episodes aimed to enhance biological homogeneity by focusing on first‐onset cases. A lifetime history of depression was excluded to minimize potential confounding by overlapping symptoms and shared biological pathways with burnout, particularly in relation to cardiovascular and stress‐related biomarkers. While there is substantial overlap between the 2 conditions, both cross‐sectionally and longitudinally,^35^ we sought to isolate the biological correlates of burnout per se, rather than those attributable to depressive pathology, thereby improving internal validity. Further exclusion criteria for both groups were familial hypercholesterolemia; diabetes; stage II hypertension; renal insufficiency; body mass index ≥35 kg/m^2^; chronic risky alcohol use; allergy to iodine‐based contrast media; contraindications for adenosine, β blockers, or nitrates; use of medication influencing blood biomarkers; and refusal of information on clinically relevant cardiac imaging findings.

Evaluation of CFR and additional data collection were conducted on the same day for all participants, except 1 who did not complete the Maslach Burnout Inventory–Human Services Survey and PHQ‐9 on the study day. For this participant, screening interview scores were used instead.

### Cardiac Imaging

A state‐of‐the‐art positron emission tomography–computed tomography scanner (Discovery MI; GE Healthcare, Waukesha, WI) with ^13^N‐ammonia as the flow tracer was used to assess MBF. To further reduce measurement bias, cardiac imaging was performed uniformly and objectively using standardized protocols. Imaging was performed during rest, adenosine‐induced hyperemia, reflecting endothelium‐independent vasodilation,^36^ and the CPT, reflecting endothelium‐dependent vasodilation.^37^ The procedure started with the intravenous injection of ^13^N‐ammonia, followed by image acquisition over 20 minutes. Adenosine was then infused intravenously at 140 μg/kg per minute for 6 minutes, with a second bolus of ^13^N‐ammonia administered 3 minutes into the infusion, and images were captured using the same protocol. After a 10‐minute interval, the CPT was conducted by immersing the participant's right foot and lower calf in ice water (4 °C) for 2 minutes. A third dose of ^13^N‐ammonia was injected 60 seconds into the CPT, followed by imaging. Dynamic, ECG‐gated and static data sets were reconstructed from each of the 3 acquisitions. Baseline and continuous measurements of heart rate, blood pressure (BP), and a 12‐lead ECG were recorded throughout. The RPP was calculated by multiplying the heart rate (in bpm) by the systolic BP (in mm Hg). The peak RPP was used as an index of the maximum myocardial workload during the stress phase and as a measure of the maximum sympathetic stimulation with the CPT.^38^ MBF at rest (RPP‐adjusted), during adenosine stress, and during the CPT was calculated using QPET software (QPET 2017.7; Cedars‐Sinai Medical Center, Los Angeles, CA). Global CFR for the left ventricle was determined by the ratio of stress‐to‐rest MBF under adenosine and CPT conditions.

### Psychometric Assessment

#### Burnout

Occupational burnout was evaluated using a validated German version of the 22‐item Maslach Burnout Inventory‐Human Services Survey, which includes 3 subscales: emotional exhaustion (9 items; score range, 0‐54), depersonalization (5 items; score range, 0–30), and personal accomplishment (8 items; score range, 0–48).^32^ Each item was rated on a 7‐point scale ranging from 0 (“never”) to 6 (“daily”). For each subscale, the item scores were summed to obtain the corresponding total scores. In this study, Cronbach's α was 0.95 for emotional exhaustion, 0.87 for depersonalization, and 0.77 for personal accomplishment.

#### Depressive Symptoms

The severity of depressive symptoms over the preceding 2 weeks was measured using the validated German version of the PHQ‐9.^39^ Participants rated the 9 items on a 4‐point Likert scale from 0 (“not at all”) to 3 (“nearly every day”). The 9 item scores were summed to obtain a PHQ‐9 total score. Higher total scores (range, 0–27) indicate more severe depressive symptoms. In this study, Cronbach's α for the PHQ‐9 total scale was 0.79.

#### Type D Personality

The validated German version of the Type D Personality Scale was used to assess TDP.^40^ The scale consists of 14 items, divided into the 2 subscales NA and SI, with 7 items each. Respondents rated their agreement with each item on a 5‐point Likert scale ranging from 0 (“not at all true”) to 4 (“completely true”). Typical items include statements such as “I often feel unhappy” (NA) and “I often feel inhibited in social interactions” (SI). For each subscale, the 7 item scores were summed to obtain total scores for NA (range, 0–28) and SI (range, 0–28). In line with the primary analytic strategy of this study, TDP was primarily operationalized dimensionally using continuous NA and SI scores and their interaction (NA×SI). For comparison with earlier studies, we also calculated a categorical TDP variable based on conventional cutoffs (NA ≥10 and SI ≥10).^41^ In this study, Cronbach's α was 0.93 for the NA scale and 0.91 for SI scale.

### Assessment of Traditional Cardiovascular Risk Factors

#### Physical Activity

Participants were asked about the frequency of engaging in sports activities that induce sweating during a typical week. Response options ranged from 0 to 7.

#### Systematic Coronary Risk Evaluation 2

This algorithm was applied as a predictor of the 10‐year risk of fatal and nonfatal CVD in individuals aged <70 year without prior CVD or diabetes.^42^ The Systematic Coronary Risk Evaluation 2 (SCORE2) incorporates age, sex, systolic BP, smoking status, total cholesterol, and high‐density lipoprotein cholesterol levels.

#### Metabolic Syndrome Factors

The number of factors to define the metabolic syndrome (MetS) in men, according to the Adult Treatment Panel III clinical identification criteria,^43^ was summed as an indicator of cardiometabolic risk severity (score range, 0–5). These 5 risk factors include abdominal obesity (waist circumference >102 cm), triglycerides ≥150 mg/dL, high‐density lipoprotein cholesterol <40 mg/dL, BP (systolic BP ≥130 mm Hg and/or diastolic BP ≥85 mm Hg), and fasting glucose ≥110 mg/dL.

BP was calculated as the average of 2 resting measurements obtained using the Arteriograph (Arteriomed GmbH, Grevenbroich, Germany). Blood lipid and glucose levels were measured from fasting samples by the Institute of Clinical Chemistry at the University Hospital Zurich.

### Data Analysis

Data analysis was conducted using IBM SPSS Statistics Version 29.0 (IBM Corp., Armonk, NY) with a significance level of *P*<0.05 (2‐tailed). BP data were missing for 2 participants, and waist circumference data for 1 participant. Missing values were imputed using the expectation–maximization algorithm. Nonnormal distributions of CFR and peak RPP values were addressed through a 2‐step transformation, preserving the original mean±SD.^44^ Group differences in categorical variables were analyzed using Pearson's χ^2^ test or Fisher's exact test, while differences in continuous variables were assessed with the Mann–Whitney *U* test.

Multivariate analyses of variance (MANOVAs) were followed by planned post hoc multivariable analyses for individual dependent variables to further explore any significant effects observed in the overall model. These analyses examined associations between TDP measures, burnout, and their interaction with endothelium‐dependent and endothelium‐independent CFR, applying 4 distinct TDP assessment approaches.^4^ The primary analytic strategy modeled TDP as a dimensional construct beginning with a continuous approach that assessed the additive main effects of the 2 subscales (NA+SI). A synergistic model then incorporated both subscale main effects and their interaction (NA+SI+NA×SI), using mean centered NA and SI total scores. Finally, if a significant synergistic effect between NA and SI was observed, a quadratic effects model was calculated to assess whether the synergistic effect was confounded by increasing NA or SI effects at higher trait scores (NA+SI+NA^2^+SI^2^). If the quadratic effects model did not reveal a significant quadratic effect, the synergistic model was reported as the final model. For completeness and comparison with previous studies, a secondary categorical approach defined TDP on the basis of high scores on both subscales (NA ≥10 and SI ≥10).

Age, SCORE2, MetS factors, and physical activity were selected as covariates on the basis of their relevance to CVD risk. However, as the effect of TDP on CFR may be partly mediated by physical activity, MetS factors, and SCORE2, we conducted additional sensitivity analyses excluding these variables to confirm the robustness of the observed associations. Particularly, if the TDP–CFR association remained stable, this would suggest no evident mediation by these factors. Depressive symptoms were excluded due to high correlation between the PHQ‐9 and Maslach Burnout Inventory–Human Services Survey total scores among the 60 participants (Spearman's *r*=0.83), and because the burnout group was partially defined based on the PHQ‐9 total score, raising multicollinearity concerns. The primary CVD risk factor model included physical activity and the SCORE2 (which accounts for age) to reduce the risk of overfitting. A confirmatory CVD risk factor model replaced the SCORE2 with age and the sum of MetS factors, while retaining physical activity (note that the confirmatory model is expected to have slightly less power due to the inclusion of an additional covariate). Multivariable analyses also explored TDP effects on peak RPP to investigate whether peak RPP (expressed per 10 000 units) was a potential mediator in the association between TDP measures and CFR. To formally test the hypothesis, we conducted a bootstrap mediation analysis using the PROCESS macro (version 4.2) in SPSS with 5000 resamples, adjusting for covariates.^45^

Mahalanobis distance indicated no multivariate outliers in the regression analyses (all *P*>0.001). Effect sizes were reported as partial η^2^ to quantify the strength of independent associations, with thresholds of 0.01, 0.06, and 0.14 representing small, medium, and large effects, corresponding to 1%, 6%, and 14% of explained variance, respectively.

## Results

### Participant Characteristics

Table 1 presents the characteristics of the 60 study participants, categorized by burnout status. Participants with burnout were significantly younger and, per the study design, had higher scores on burnout subscales and more severe depressive symptoms compared with those in the nonburnout group. The burnout group also showed significantly elevated levels of NA and SI. Among the 14 participants (23.3%) meeting categorical criteria for TDP, 13 (92.9%) also experienced burnout. For reference and comparability with prior studies, participant characteristics by categorical TDP status are shown in the Table S1.

**Table 1:** Characteristics of the 60 Study Participants by Burnout Groups

Variable	Burnout group (n=30)	Nonburnout group (n=30)	*P* value
Age, y, mean±SD	46.77±10.56	52.93±7.48	0.022
Body mass index, kg/m^2^, mean±SD	25.63±3.09	24.35±2.72	0.080
Physical activity, times/wk, mean±SD	1.99±1.62	2.67±1.92	0.160
Cardiovascular risk score, %, mean±SD	3.11±1.89	3.47±1.89	0.258
Metabolic syndrome factors, mean±SD	1.10±1.16	0.67±0.88	0.137
Fasting glucose ≥110 mg/dL, n (%)	0 (0)	2 (6.7)	0.492
Triglycerides ≥150 mg/dL, n (%)	9 (30.0)	3 (10.0)	0.053
HDL cholesterol <40 mg/dL, n (%)	5 (16.7)	0 (0)	0.052
Waist circumference >102 cm, n (%)	5 (16.7)	3 (10.0)	0.706
SBP ≥130 mm Hg and/or DBP ≥85 mm Hg, n (%)	14 (46.7)	12 (40.0)	0.602
Emotional exhaustion, score, mean±SD	29.17±7.13	6.67±3.99	<0.001
Depersonalization, score, mean±SD	11.33±7.00	3.07±3.60	<0.001
Low personal accomplishment, score, mean±SD	12.03±6.74	5.67±4.37	<0.001
Negative affectivity, score, mean±SD	11.77±5.72	4.17±4.27	<0.001
Social inhibition, score, mean±SD	11.73±6.34	7.27±5.50	0.006
Depressive symptoms, score, mean±SD	7.40±3.13	2.20±1.97	<0.001
Myocardial blood flow, rest, mL/g per min, mean±SD	0.64±0.13	0.66±0.10	0.429
Coronary flow reserve (adenosine), mean±SD	4.31±1.43	4.58±1.09	0.214
Peak rate‐pressure product (adenosine), mean±SD	12 245±2740	10 753±3097	0.053
Coronary flow reserve (cold pressor test), mean±SD	1.50±0.52	1.48±0.45	0.953
Peak rate‐pressure product (cold pressor test), mean±SD	9829±2306	9681±3052	0.469

Normalized values are given for measures of microvascular function. The peak rate–pressure product indicates the maximum value measured during intravenous application of adenosine or the cold pressor test. Group differences were analyzed using the Mann–Whitney *U* test for continuous variables and the Pearson χ^2^ test or Fisher's exact test for categorical variables, as appropriate. DBP indicates diastolic blood pressure; HDL, high‐density lipoprotein; and SBP, systolic blood pressure.

### Interactions Between Burnout and TDP Measures in Relation to CFR

First, we conducted a MANOVA to assess whether burnout moderates the effect of TDP on the dependent variables—endothelium‐dependent CFR and endothelium‐independent CFR considered together—using 4 different methods to measure TDP. To avoid overfitting, additional covariates were not included in these models. Burnout showed no significant interaction with NA scores (*P*=0.82) or SI scores (*P*=0.17) after adjusting for the main effects of burnout, NA scores, and SI scores. Furthermore, after adjusting for the main effects of burnout, NA, SI, NA^2^, SI^2^, as well as for the burnout‐by‐NA and burnout‐by‐SI interactions, neither the burnout‐by‐NA^2^ interaction (*P*=0.30) nor the burnout‐by‐SI^2^ interaction (*P*=0.46) reached significance. Additionally, no significant 3‐way interaction among burnout, NA scores, and SI scores was detected after adjusting for main effects and 2‐way interactions between these variables (*P*=0.52). Taken together, these results provided no evidence for burnout as a moderator in the relationship between TDP and CFR. Interaction analyses involving categorical TDP and burnout were not appropriate, as only 1 participant had TDP without concurrent burnout, precluding meaningful statistical evaluation.

### Independent Associations of Type D Measures and Burnout With CFR

As the above results suggested that burnout is not an evident moderator of the relationship between TDP and CFR, subsequent analyses focused on examining the independent associations of the different TDP measures and burnout with CFR, adjusting for covariates. These included physical activity and either the SCORE2 (primary CVD risk factor model) or age together with the number of MetS factors (confirmatory CVD risk factor model). Notably, burnout did not show a significant independent association with CFR in these analyses (statistics not reported).

#### Continuous Method

A significant interaction between continuous NA and SI scores, for CFR was observed in MANOVA, after adjusting only for the main effects of continuous NA and SI scores (F [2,55]=4.831, *P*=0.012; partial η^2^=0.149). The interaction remained significant after additional adjustment for burnout, physical activity, and either SCORE2 (F [2, 52]=5.361, *P*=0.008; partial η^2^=0.171) or age and the sum of MetS factors (F [2, 51]=5.057, *P*=0.010; partial η^2^=0.165). In sensitivity analyses, removing physical activity, SCORE2, and MetS factors from the respective models did not meaningfully alter the effect size of the interaction, which remained significant (F [2, 54]=5.638, *P*=0.005; partial η^2^=0.177; and F [2, 53]=5.777, *P*=0.005; partial η^2^=0.179, respectively). This suggests that these covariates did not substantially mediate the observed association.

In addition, there were no additive effects of continuous NA and SI scores in MANOVA, either with or without covariate adjustment (*P*>0.15). Similarly, the MANOVA revealed no quadratic effects of NA and SI scores after adjusting for continuous NA and SI scores, regardless of covariate adjustment (*P*>0.08).

#### Categorical Method

There was no significant multivariate association between TDP (both NA and SI scores ≥10) and the dependent variables, endothelium‐dependent and endothelium‐independent CFR, considered as a set, regardless of covariate adjustment (*P*>0.67).

### Multivariable Analysis of the Type D Effect on CFR

Table 2 presents the follow‐up multivariable analysis of the significant multivariate model, probing for a synergistic effect of continuous NA and SI scores on each CFR measure. A significant NA×SI interaction on endothelium‐dependent CFR was observed after adjusting only for the main effects of continuous NA and SI scores (B=0.003 [95% CI, 0.0001–0.006]; *P*=0.049; partial η^2^=0.067). This interaction remained significant after additional covariate adjustment in both the primary (B=0.003 [95% CI, 0.00008–0.006]; *P*=0.044; partial η^2^=0.074) and confirmatory CVD risk factor model (B=0.003 [95% CI, 0.0003–0.006]; *P*=0.030; partial η^2^=0.087). When physical activity, SCORE2, and the MetS factors were removed from the respective models in sensitivity analyses, the effect size of the association remained largely unchanged and significant (B=0.003 [95% CI, 0.0002–0.006]; *P*=0.036; partial η^2^=0.077; and B=0.003 [95% CI, 0.0002–0.006]; *P*=0.038; partial η^2^=0.077, respectively). This suggests that these covariates did not substantially mediate the observed association.

**Table 2:** Multivariable Association Between the NA×SI Interaction and CFR

Covariates	Endothelium‐dependent CFR (cold pressor test)		Endothelium‐independent CFR (adenosine)	
Burnout	0.214 (−0.100 to 0.528)	0.312 (−0.013 to 0.637)	−0.266 (−1.106 to 0.574)	−0.143 (−1.023 to 0.736)
SCORE2	0.019 (−0.051 to 0.089)		−0.130 (−0.318 to 0.057)	
Age		0.008 (−0.007 to 0.022)		−0.013 (−0.053 to 0.027)
MetS factors		−0.093 (−0.217 to 0.032)		−0.286 (−0.623 to 0.050)
Physical activity	−0.010 (−0.084 to 0.064)	−0.014 (−0.088 to 0.061)	0.113 (−0.084 to 0.311)	0.113 (−0.089 to 0.314)
NA	−0.031 (−0.060 to −0.002)*	−0.032 (−0.060 to −0.003)*	0.028 (−0.050 to 0.105)	0.025 (−0.052 to 0.103)
SI	0.001 (−0.021 to 0.024)	−0.003 (−0.026 to 0.020)	−0.028 (−0.089 to 0.033)	−0.034 (−0.096 to 0.028)
NA×SI	0.003 (0.00008 to 0.006)*	0.003 (0.0003 to 0.006)*	−0.004 (−0.012 to 0.003)	−0.003 (−0.011 to 0.004)

Values are unstandardized B coefficients with 95% CIs. All independent variables were entered simultaneously in a single block. Mahalanobis distance indicated no outlier. CFR indicates coronary flow reserve; MetS, metabolic syndrome; NA, negative affectivity; SCORE2, Systematic Coronary Risk Evaluation 2; and SI, social inhibition.

* *P*<0.05.

**Figure 2:**
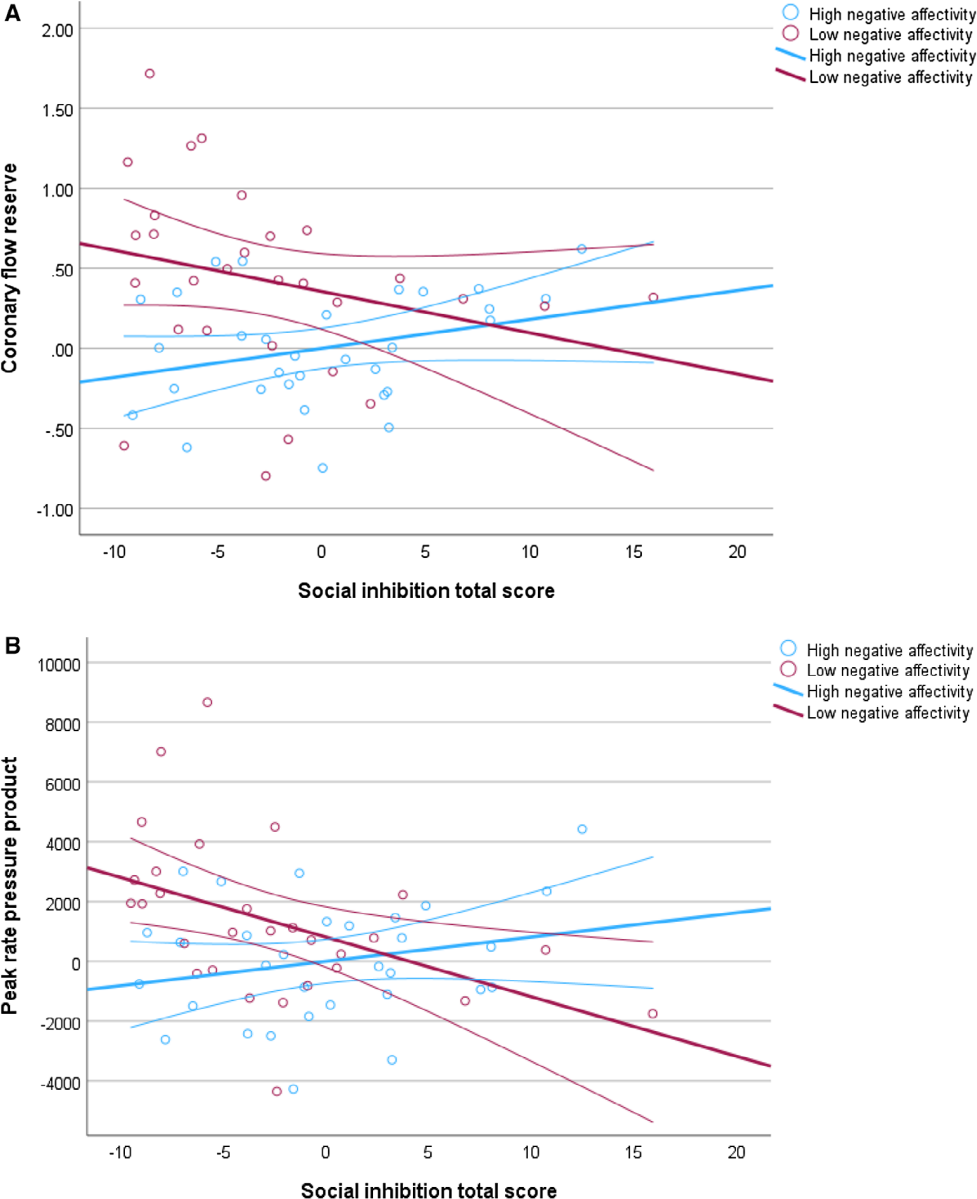
Visualization of the significant negative affectivity‐by‐social inhibition interaction for (A) endothelium‐dependent coronary flow reserve and (B) peak rate pressure product with the cold pressor test. The partial regression plots with fit lines (95% CIs) illustrate the multivariable associations between social inhibition scores (continuous) and endothelium‐dependent coronary flow reserve (**A**) and peak rate pressure product (**B**) in response to the cold pressor test, stratified by participants with high vs low negative affectivity scores (median split). Analyses were adjusted for burnout (yes/no), physical activity, and the Systematic Coronary Risk Evaluation 2.

The positive B coefficients for the interactions indicated a synergistic effect such that the effect of NA on endothelium‐dependent CFR increases with higher SI scores and vice versa, independent of the included covariates. However, visualization of the significant interaction using a median split on NA and SI scores revealed a more complex relationship than pure synergy. On the one hand, if NA was high (NA scores, 7–25; N=31), endothelium‐dependent CFR increased with higher SI scores, compatible with a positive (ie synergistic) effect of NA and SI on CFR. On the other hand, when NA was low (NA scores, 0–6; N=29), there was a negative relationship between SI and CFR such that CFR decreased with higher SI scores (Figure 2A). Likewise, when SI was high (SI scores, 9–25; N=29), there was an increase in CFR with higher NA scores, but when SI was low (SI scores, 0–8; N=31), CFR decreased with higher NA scores. A completely synergistic effect would require all regression curves to be nonnegative for both low and high SI or NA scores, with a stronger positive association with endothelium‐dependent CFR for higher than lower scores.

No significant interaction between NA and SI was observed for endothelium‐independent CFR (*P*>0.25). Additionally, none of the covariates demonstrated significant independent associations with CFR in either model.

### Peak Rate Pressure Product as a Possible Explanation for the Significant Type D Effect

A multivariable analysis, adjusting only for the main effects of NA and SI, showed a significant association between the NA×SI interaction and peak RPP in response to the CPT (B=0.002 [95% CI, 0.0002–0.003]; *P*=0.025; partial η^2^=0.087). The significance of the interaction was maintained with additional adjustment for burnout, physical activity, and SCORE2 (B=0.002 [95% CI, 0.0002–0.003]; *P*=0.022; partial η^2^=0.095). A similar result was found when controlling for age and the sum of MetS factors instead of SCORE2 (B=0.001 [95% CI, −0.00001 to 0.003]; *P*=0.052; partial η^2^=0.070).

Visualization of the significant NA×SI interaction revealed a pattern of relationships with peak RPP that was partly congruent with the above‐reported pattern for CFR. Specifically, when NA was high, peak RPP increased with higher SI scores, whereas when NA was low, peak RPP decreased with higher SI scores (Figure 2B). However, peak RPP decreased with higher NA scores regardless of whether SI was high or low, although the decrease was less pronounced when SI was high. Among the covariates, greater physical activity was independently associated with lower peak RPP (*P*=0.002; partial η^2^=0.165; *P*=0.004; partial η^2^=0.151), while higher SCORE2 (*P*=0.003; partial η^2^=0.159) and a greater sum of MetS factors (*P*=0.008; partial η^2^=0.129) were independently associated with higher peak RPP in their respective models. No significant independent association was observed between age and peak RPP (*P*=0.15).

**Figure 3:**
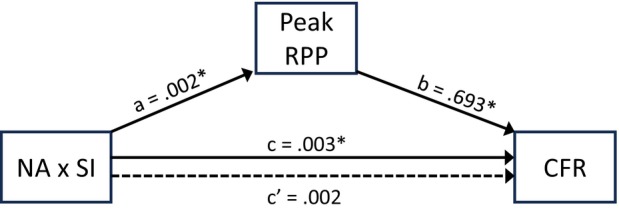
Peak rate–pressure product mediates the NA×SI interaction effect on endothelium‐dependent coronary flow reserve. The figure visualizes the mediation model, accounting for burnout (yes/no), physical activity, the SCORE2, and the main effects of the continuous scores for NA and SI as covariates. a=effect of the NA×SI interaction on peak RPP. b=effect of peak RPP on endothelium‐dependent CFR. c=effect of the NA×SI interaction on CFR (without peak RPP in model). c′=effect of the NA×SI I interaction on CFR (with peak RPP in model). a×b=indirect effect of the NA×SI interaction on CFR through RPP. *Denotes a significant association (all *P*<0.05). CFR indicates coronary flow reserve; NA, negative affectivity; RPP, rate–pressure product; SCORE2, Systematic Coronary Risk Evaluation 2; and SI, social inhibition.

When peak RPP was included as a covariate in the above multivariable analysis, the previously significant association between the NA×SI interaction and endothelium‐dependent CFR became nonsignificant (primary model with SCORE 2: B=0.002 [95% CI, −0.001 to 0.005]; *P*=0.21; confirmatory model with age and MetS factors: B=0.002 [95% CI, −0.001 to 0.005]; *P*=0.15). However, peak RPP now emerged as a significant predictor of endothelium‐dependent CFR. For a 10 000‐unit increase in peak RPP, CFR increased by 0.693 (95% CI, 0.142–1.244; *P*=0.015) in the model adjusted for SCORE2, and by 0.871 (95% CI, 0.348–1.394; *P*=0.002) in the model adjusted for age and the sum of MetS factors. This suggests that peak sympathetic stimulation during the CPT mediated the type D effect on endothelium‐dependent CFR (Figure 3). In the model adjusted for SCORE2, formal mediation analysis confirmed a significant indirect effect of the NA×SI interaction on endothelium‐dependent CFR via peak RPP (B=0.001 [95% CI, 0.00003–0.003]), with 37.9% of the total effect mediated (standardized indirect effect=0.108 [95% CI, 0.003–0.264]).

## Discussion

In our sample of practicing male physicians from Switzerland, 23.3% met TDP criteria (scores ≥10 on both NA and SI subscales). This prevalence aligns with the 22.2% reported in a representative German population‐based study^8^ and the 28.5% among Belgian hospital physicians,^23^ supporting the external validity of the observed distribution. TDP co‐occurred with burnout in 92.9% of cases, consistent with findings that hospital physicians with TDP are 7 times more likely to experience burnout.^23^ As a result of this pronounced co‐occurrence, meaningful moderation analysis was precluded, particularly in the categorical approach. Even in dimensional models, no moderating effect of burnout was detected. This limitation could not have been anticipated at the design stage, when we had planned to examine burnout as a moderator of the TDP–CFR association. Consequently, the findings are uninformative with respect to the male physician population and inconclusive regarding whether burnout moderates the association between TDP and CFR in the general population.

The main finding of our study is a significant interaction effect between NA and SI, the core components of TDP, on CFR during the CPT. While the interaction effect was positive, the relationship was more complex than pure synergy. Specifically, CFR showed a positive relationship with SI only when NA was high, and with NA only when SI was high. Although the strict requirement for all conditional regression curves to be positive is rarely met in other TDP studies reporting positive interaction effects,^46^ we interpret this finding as indicative of a synergistic effect, where higher NA and SI scores amplify their combined impact, enhancing endothelium‐dependent CFR during sympathetic stimulation.

Underscoring the robustness of this interpretation, the main finding was independent of physicians' burnout status and traditional CVD risk factors, including age, physical activity, and metabolic variables, across 2 CVD risk factor models. There was little evidence that physical activity, SCORE2, or the sum of MetS factors acted as potential mediators of the observed association between TDP and CFR. Notably, the synergistic effect uniquely explained ≈8% of the variance in CFR, corresponding to a medium effect size, thus indicating clinical relevance. The lack of an additive effect of NA and SI suggests that the 2 subscales were not independently associated with CFR, while the absence of quadratic effects indicates that the synergistic relationship remains consistent across a range of scores, rather than being driven by increasing NA or SI effects at higher trait scores.^30^ There was also no significant association between TDP, as defined by the categorical approach (high scores in both NA and SI), and CFR, supporting the interpretation that the relationship between NA, SI, and CFR operates on a continuum rather than strict dichotomies. Future research should prioritize examining NA and SI as continuous variables to better capture their interactive effects on cardiovascular and other health outcomes.

Regarding a potential mechanism underlying the synergistic effect of NA and SI on endothelium‐dependent CFR, increase in peak RPP during sympathetic stimulation emerged as a plausible mediator with a medium effect size, highlighting its clinical relevance. The variance in peak RPP explained by the NA×SI interaction was about half that of traditional CVD risk factors known to influence sympathetic activity.^47^, ^48^ Specifically, greater physical activity correlated with lower peak RPP, while higher SCORE2 values and more MetS factors were linked to elevated peak RPP. The formal mediation analysis confirmed a significant indirect effect of the NA×SI interaction on endothelium‐dependent CFR via peak RPP, accounting for ≈38% of the total effect.

CFR during adenosine‐induced vasodilation that is independent of sympathetic pathways showed no association with TDP, suggesting that TDP is not linked to reduced endothelium‐independent CFR, a marker of major adverse cardiovascular event risk.^17^ However, the observed increase in CFR during the CPT should not be viewed as protective but rather as an exaggerated response driven by the NA–SI interaction. This may indicate instability in coronary regulation, with chronic psychosocial stress from TDP traits sensitizing sympathetic responsiveness.^49^ In our participants still without clinical CVD, this exaggerated CFR could reflect a compensatory early‐stage response, potentially indicative of maladaptive hyperemia, leading to vascular remodeling, endothelial dysfunction, and heightened atherosclerotic risk over time. This aligns with the concept of allostatic load, where maladaptive responses to repeated stressors compromise long‐term health.^50^ Future longitudinal studies should test this hypothesized sequence of events and explore sex‐related differences, which were beyond the scope of our research. For instance, a meta‐analysis found TDP linked to lower BP reactivity to acute stress in women,^46^ while men with TDP showed heightened cardiac sympathetic responses during the CPT compared with women and men without TDP,^16^ aligning with our findings.

Our findings have potential clinical implications. Interventions targeting sympathetic activity in individuals with TDP, such as through cognitive–behavioral therapy,^51^ stress management and physical activity promotion,^52^ or mindfulness practices^53^ may help reduce cardiovascular risk. Tailored approaches should address the specific emotional and social challenges faced by type D individuals, including the tendency of those with high SI to suppress distress (related to NA), which can exacerbate cardiac sympathetic responses. Rather than focusing exclusively on individuals meeting strict TDP criteria, interventions should address varying levels of NA and SI. Combining cognitive–behavioral strategies for NA with social skills training for SI could prove particularly effective. Additionally, psychotherapy integrated with biofeedback may help manage both emotional distress and exaggerated cardiac sympathetic responses. Programs designed to reduce rumination (associated with NA) and promote positive social interactions (countering SI) could further support cardiovascular health.

The strengths of this study include robust cardiac imaging techniques to objectively measure CFR and participant matching on key demographic and clinical factors, reducing potential confounding. Multiple approaches to assess TDP add depth, particularly enhancing the understanding of NA×SI interaction effects. However, several limitations should be considered. The cross‐sectional design limits causal inferences and despite careful matching and statistical adjustments, residual confounding from unmeasured variables (eg, sleep quality or diet) remains possible, likely weakening true associations. Our quota sampling among physicians might have introduced selection bias, potentially underestimating associations if severely distressed individuals avoided participation. The focus on male physicians also reduces generalizability to other populations, such as women or individuals in different occupations. The exclusion of participants with depression or a prior episode of burnout resulted in a selective burnout sample, further limiting generalizability. Additionally, exclusion of known CAD focused the sample on individuals without diagnosed atherosclerosis, although the presence of subclinical disease cannot be ruled out. Because data collection partially overlapped with the SARS‐CoV‐2 pandemic, a time of heightened job‐related stress among Swiss health care workers,^54^ our findings may not fully reflect typical conditions and should be validated outside the pandemic context. The relatively small sample size may have limited the detection of subtle associations or main effects, while the high co‐occurrence of burnout and TDP substantially limited the feasibility of meaningful moderation analysis. The reliance on self‐reported measures for psychosocial traits and burnout introduces potential reporting bias, likely attenuating observed relationships. Moreover, dichotomizing the continuum of burnout experience, a naturally continuous construct lacking well‐established diagnostic thresholds,^25^ may have contributed to our finding of no significant relationship between burnout and CFR.

In conclusion, the findings of this study propose a synergistic effect of TDP traits on coronary microvascular function, mediated by sympathetic activation in male physicians. Future research should validate this effect in diverse populations and explore interventions addressing NA and SI to reduce sympathetic activation and mitigate long‐term risk of CAD.

## Sources of Funding

Financial support for the study was provided by an institutional grant from the University of Zurich, Switzerland, provided to R.v.K.

## Disclosures

None.

## Supporting information

Table S1
