# Supplementary material for: Synergistic Effects of Type D (“Distressed”) Personality Traits on Coronary Microvascular Function in Male Physicians With Occupational Burnout: A Cross‐Sectional Study
Source: J Am Heart Assoc. 2025 Jul 29;14(15):e041273. doi: 10.1161/JAHA.125.041273 (PMC12450003; doi:10.1161/JAHA.125.041273)
Supplement: Supplementary file 1 — Table S1 [file JAH3-14-e041273-s001.pdf]

Supplemental Material

Table S1.

Characteristics of the 60 study participants by categorical type D personality status

Variable	TDP group (n=14)	Non-TDP group (n=46)	P
Age, years	44.43 (9.28)	51.50 (9.12)	0.015
Body mass index, kg/m^2	24.58 (2.68)	25.11 (3.05)	0.618
Physical activity, times/week	1.77 (1.68)	2.50 (1.81)	0.212
Cardiovascular risk score, %	2.54 (1.73)	3.52 (1.89)	0.052
Metabolic syndrome factors, n	0.93 (1.00)	0.87 (1.07)	0.728
Fasting glucose ≥110 mg/dL, n (%)	0 (0)	2 (4.3)	1.000
Triglycerides ≥150 mg/dL, n (%)	4 (28.6)	8 (17.4)	0.448
HDL cholesterol <40mg/dL, n (%)	2 (14.3)	3 (6.5)	0.582
Waist circumference >102 cm, n (%)	1 (7.1)	7 (15.2)	0.667
SBP ≥130 mmHg and/or DBP ≥85 mmHg, n (%)	6 (42.9)	20 (43.5)	0.967
Burnout, n (%)	13 (92.9)	17 (37.0)	<0.001
Emotional exhaustion, score	32.14 (7.94)	13.59 (1055)	<0.001
Depersonalization, score	13.07 (7.36)	5.41 (5.75)	<0.001
Low personal accomplishment, score	15.79 (7.62)	6.74 (4.34)	<0.001
Negative affectivity, score	16.07 (4.34)	5.50 (4.47)	<0.001
Social inhibition, score	16.50 (5.19)	7.37 (4.94)	<0.001
Depressive symptoms, score	8.21 (2.83)	3.76 (3.28)	<0.001
Myocardial blood flow, rest, mL/g/min	0.66 (0.15)	0.65 (0.10)	0.930
Coronary flow reserve (adenosine)	4.18 (1.22)	4.52 (1.28)	0.354
Peak rate-pressure product (adenosine)	11958 (2202)	11359 (3206)	0.368
Coronary flow reserve (cold pressor test)	1.43 (0.37)	1.51 (0.51)	0.662
Peak rate-pressure product (cold pressor test)	9422 (2230)	9857 (2820)	0.793

DBP, diastolic blood pressure; HDL, high-density lipoprotein; SBP, systolic blood pressure; TDP, type D personality (negative affectivity score > 10 plus social inhibition score >10).

Values are given as mean with standard deviation (in parentheses) or absolute numbers with percentages (in parentheses). Normalized values are given for measures of microvascular function. The peak rate-pressure product indicates the maximum value measured during intravenous application of adenosine or the cold pressor test. Group differences were analyzed using the Mann-Whitney U test for continuous variables and the Pearson chi-square test or Fisher's exact test for categorical variables, as appropriate.
